# Identification of the circRNA–miRNA–mRNA regulatory network in osteoarthritis using bioinformatics analysis

**DOI:** 10.3389/fgene.2022.994163

**Published:** 2022-09-16

**Authors:** Wen-Bin Xu, Vit Kotheeranurak, Huang-Lin Zhang, Jin-Yi Feng, Jing-Wei Liu, Chien-Min Chen, Guang-Xun Lin, Gang Rui

**Affiliations:** ^1^ Department of Orthopedics, The First Affiliated Hospital of Xiamen University, School of Medicine, Xiamen University, Xiamen, China; ^2^ Department of Orthopedics, Faculty of Medicine, Chulalongkorn University, and King Chulalongkorn Memorial Hospital, Bangkok, Thailand; ^3^ Center of Excellence in Biomechanics and Innovative Spine Surgery, Chulalongkorn University, Bangkok, Thailand; ^4^ The Third Clinical Medical College, Fujian Medical University, Fuzhou, Fujian, China; ^5^ Division of Neurosurgery, Department of Surgery, Changhua Christian Hospital, Changhua, Taiwan; ^6^ Department of Leisure Industry Management, National Chin-Yi University of Technology, Taichung, Taiwan; ^7^ School of Medicine, Kaohsiung Medical University, Kaohsiung, Taiwan

**Keywords:** osteoarthritis, circRNA, ceRNA, GEO, bioinformatics analysis

## Abstract

**Background:** Osteoarthritis (OA) is a degenerative joint disease that seriously affects the quality of people. Unfortunately, the pathogenesis of OA has not been fully known. Therefore, this study aimed to construct a ceRNA regulatory network related to OA to explore the pathogenesis of OA.

**Methods:** Differentially expressed circRNAs (DEcircRNAs), microRNAs (DEmiRNAs), and mRNAs (DEmRNAs) were obtained from the Gene Expression Omnibus microarray data (GSE175959, GSE105027, and GSE169077). The miRNA response elements and target mRNAs were identified using bioinformatics approaches. Additionally, a circRNA–miRNA–mRNA network was established using Cytoscape version 3.8.0. Gene Ontology (GO) and Kyoto Encyclopedia of Genes and Genomes (KEGG) analyses of mRNAs in the network were conducted to explore the possible mechanisms underlying OA development. Protein–protein interaction (PPI) analysis was performed to determine the hub genes. Based on the hub genes, a sub network was constructed using Cytoscape 3.8.0 version. Finally, connectivity map (CMap) and drug–gene interaction database (DGIdb) analyses were performed to identify the potential therapeutic targets for OA.

**Results:** Altogether, five DEcircRNAs, 89 DEmiRNAs, and 345 DEmRNAs were identified. Moreover, a circRNA–miRNA–mRNA network was established using three circRNAs, seven miRNAs, and 37 mRNAs. GO and KEGG analyses demonstrated that the mRNAs in the network could be related to the occurrence and development of OA. PPI analysis was performed and six key genes, namely serpin family H member 1 [*SERPINH1*], collagen type VIII alpha 2 chain [*COL8A2*], collagen type XV alpha 1 chain [*COL15A1*], collagen type VI alpha 3 chain [*COL6A3*], collagen type V alpha 1 chain [*COL5A1*], and collagen type XI alpha 1 chain [*COL11A1*], were identified. Furthermore, a circRNA–miRNA–hub gene subnetwork was established in accordance with two circRNAs (hsa_circ_0075320 and hsa_circ_0051428), two miRNAs (hsa-miR-6124 and hsa-miR-1207-5p), and six hub genes (*COL11A1*, *SERPINH1*, *COL6A3*, *COL5A1*, *COL8A2*, and *COL15A1*). Finally, three chemicals (noscapine, diazepam, and TG100-115) based on CMap analysis and two drugs (collagenase *Clostridium histolyticum* and ocriplasmin) based on DGIdb were discovered as potential treatment options for OA.

**Conclusion:** This study presents novel perspectives on the pathogenesis and treatment of OA based on circRNA-related competitive endogenous RNA regulatory networks.

## Introduction

Osteoarthritis (OA) is a common arthrosis illness that may result in joint dysfunction, severely affecting the patient’s quality of life and increasing the social and economic burden (Cross et al., 2014; Kiadaliri et al., 2018). The prevalence of OA is high in middle-aged and elderly populations, and more than 50% of patients with knee pain are diagnosed with OA (Tang et al., 2016; Lespasio et al., 2017). However, the pathogenesis of OA is not yet fully understood. Therefore, understanding the pathogenesis of OA is essential to identify effective diagnostic and therapeutic targets.

Circular RNA (circRNAs) are neoteric noncoding RNAs (ncRNAs) with a circular form (Petkovic and Müller, 2015; Qu et al., 2015). In the absence of 5′ caps and 3′ tails, circRNAs are not influenced by exonucleases and become relatively stable (Li et al., 2015). CircRNAs, characterized by their specificity (Salzman et al., 2013) and high conservation (Vidal et al., 2016) are considered to play significant roles in various diseases (Kristensen et al., 2018).

Competing endogenous RNAs (ceRNAs) serve as transcripts for microRNA (miRNA) sponges and mutually regulate each other by binding to miRNAs (Qi et al., 2015). Currently, circRNAs are abundant in conserved miRNA response elements (MREs), which have emerged as novel targets in the ceRNA family (Zhong et al., 2018). Some circRNAs are involved in the occurrence and progression of various tumors *via* the ceRNA regulatory process (Wei et al., 2018; Xiong et al., 2018). However, whether circRNAs can also regulate the occurrence of OA via the ceRNA mechanism remains to be investigated.

**FIGURE 1:**
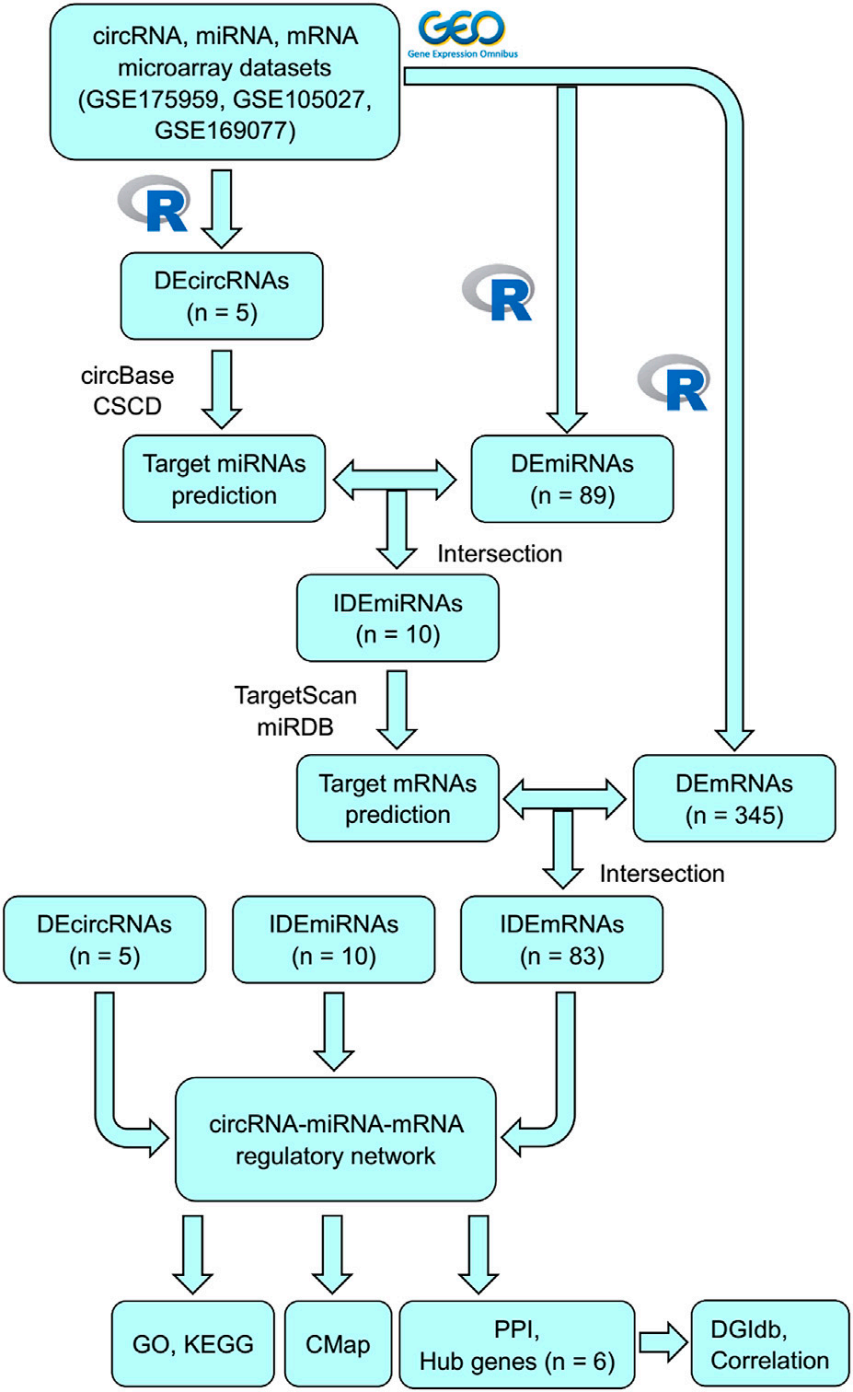
Flowchart of this study.

In this study, some unknown circRNAs and their potential regulatory mechanisms in OA were analyzed using bioinformatic analysis. Figure 1 shows the flowchart of the entire procedure. First, differentially expressed circRNAs (DEcircRNAs), miRNAs (DEmiRNAs), and mRNAs (DEmRNAs) were obtained from the Gene Expression Omnibus (GEO) datasets. To characterize the role of DEcircRNAs as ceRNAs in OA, MREs and target mRNAs were predicted and a circRNA–miRNA–mRNA network was constructed. Gene Ontology (GO) and Kyoto Encyclopedia of Genes and Genomes (KEGG) analyses were performed according to the mRNAs identified in the network to explore the possible mechanisms associated with OA. Subsequently, protein–protein interactions (PPIs) were used to identify related key mRNAs. Finally, connectivity map (CMap) and drug–gene interactions database (DGIdb) analyses were performed to identify potential compounds or drugs to treat OA.

## Materials and methods

### Microarray data

Microarray data were extracted from the GEO database (Barrett et al., 2013). The circRNA expression profile was acquired from GSE175959 (three OA and three healthy knee tissues), the miRNA expression profile was obtained from GSE105027 (12 pairs of OA knee serum and healthy knee serum), and the mRNA expression profile was obtained from GSE169077 (five OA and six healthy knee tissues). Ethical approval was not required for our research because the data from GEO database are publicly available.

### Differential expression analysis

The specific process was as follows: 1) The platform files and series of matrix files of GSE175959, GSE105027, and GSE169077 were downloaded, and the corresponding probe names were transformed into international standard names (circRNA name, miRNA name, and gene symbol) by utilizing the practical extraction and report language (Perl); 2) The normalizeBetweenArrays package was used to normalize the data; 3) DEcircRNAs, DEmiRNAs, and DEmRNAs were selected using the Bioconductor Limma package, with the criteria as adjusted *p*-value < 0.05 and |log2(fold-change)| > 1.00(Ritchie et al., 2015); and 4) Heatmaps of DEcircRNAs, DEmiRNAs, and DEmRNAs were generated using the R package pheatmap.

### Prediction of miRNA response elements

The circBase (Glažar et al., 2014) and cancer-specific circRNAs database (CSCD) (Xia et al., 2018) were used to predict the target miRNA binding sites of DEcircRNAs. Furthermore, we analyzed the interactions of the target miRNAs of DEcircRNAs and DEmiRNAs, which were called as IDEmiRNAs.

### Forecasting of target mRNAs

IDEmiRNA–mRNA interactions were predicted according to the TargetScan (Agarwal et al., 2015) and miRDB databases (Wong and Wang, 2015). Only mRNAs that appeared in both databases were chosen and intersected with the DEmRNAs to obtain candidate target mRNAs, which were designated as IDEmRNAs.

### Construction of the circRNA–miRNA–mRNA network

circRNAs, IDEmiRNAs, and IDEmRNAs were selected to establish a circRNA–miRNA–mRNA regulatory network. We used Cytoscape 3.8.0 version to view this network (Su et al., 2014).

### Functional enrichment analysis

Before analysis, the gene symbols of mRNAs in the network were transformed into entrezIDs using R. Then, GO and KEGG analyses were implemented using the clusterProfiler package (Kanehisa and Goto, 2000; Yu et al., 2012). Both the *p*-value and q-values of GO analysis were <0.05, and the *p*-value of the KEGG analysis was <0.05.

### Connectivity map analysis

CMap is a gene expression profiling database that uses the L1000 analysis platform to explore the network of interactions among drug/small-molecule compounds, genes, and disease states (Lamb et al., 2006). CMap analysis according to upregulated mRNAs in the ceRNA network was performed to identify candidate compounds for OA treatment. The connectivity scores ranged from –100 to 100, which was adapted to indicate closeness between the genes and compounds, with a positive score indicating a positive correlation with the uploaded genes and a negative score indicating a negative correlation with the uploaded genes.

### Construction of the protein–protein interaction network

According to the mRNAs discovered in the network, PPI analysis was performed using the Search Tool for the Retrieval of Interacting Genes/Proteins (STRING) (Szklarczyk et al., 2017). We then performed a visual analysis of the PPI network using Cytoscape 3.8.0 (Su et al., 2014). In addition, we also used the molecular complex detection (MCODE) (Bader and Hogue, 2003) plugin in Cytoscape software to defect essential modules and hub genes with the criterion of default parameters.

### Correlation network analysis

Correlation network analysis for hub genes was conducted using R software (igraph package). The correlation coefficient greater than 0.2 was considered to be relevant.

### Drug–gene interaction analysis

Drug–gene interaction analysis of hub genes was performed using DGIdb (https://dgidb.org/) (Griffith et al., 2013). DGIdb is an open-source project that provides information about genes associated with known or potential drugs. We uploaded the significant genes to DGIdb for matching with known drugs to identify the potential targets for OA treatment.

## Results

### Identification of DEcircRNAs, DEmiRNAs, and DEmRNAs

**FIGURE 2:**
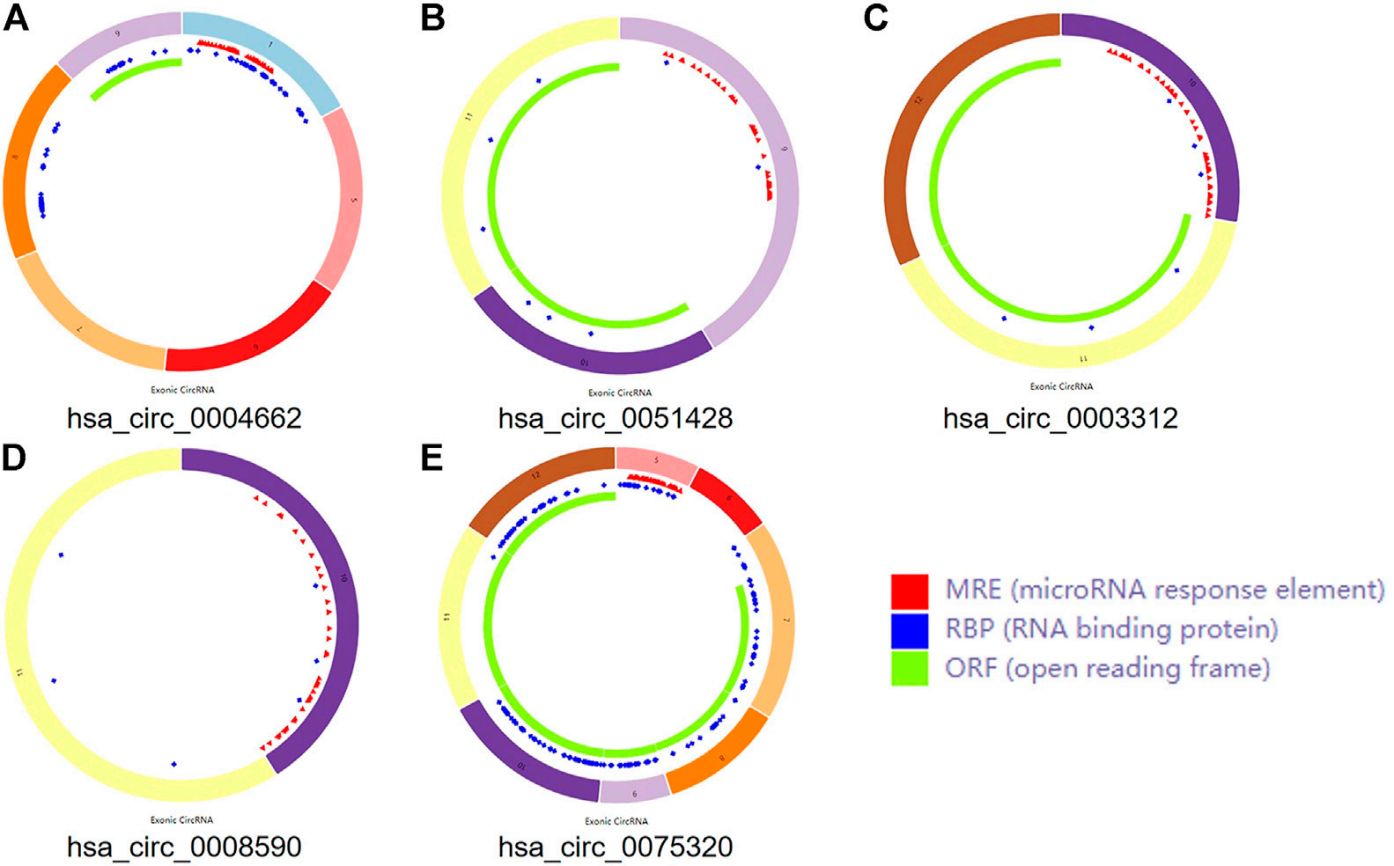
The structural patterns of the five circRNAs. **(A)** hsa_circ_0004662. **(B)** hsa_circ_0051428. **(C)** hsa_circ_0003312. **(D)** hsa_circ_0008590. **(E)** hsa_circ_0075320.

Three microarray datasets, GSE175959, GSE105027, and GSE169077, for circRNAs, miRNAs, and mRNAs, respectively, were used in our study. Based on the criteria set in advance [adjusted *p*-value < 0.05 and |log2(fold-change) | > 1.00], five DEcircRNAs (all upregulated) were identified in the GSE175959 dataset (Supplementary Figure S1A), 89 DEmiRNAs (all downregulated) were identified from the GSE105027 dataset (Supplementary Figure S1B), and 345 DEmRNAs (194 downregulated and 151 upregulated) were identified in the GSE169077 dataset (Supplementary Figure S1C). Supplementary Table S1 shows the basic information of these five DEcircRNAs, hsa_circ_0004662, hsa_circ_0051428, hsa_circ_0003312, hsa_circ_0008590, and hsa_circ_0075320. The circular structural styles are shown in Figure 2.

### Identification of IDEmiRNAs

Some circRNAs act as sponges and play an important role in trapping miRNAs in non-tumorous tissues. To determine whether the five circRNAs showed similar functions in OA, MREs were predicted according to CSCD. A total of 239 MREs were identified after excluding duplicate target miRNAs. Ten significant IDEmiRNAs were obtained by intersecting 239 MREs with 89 DEmiRNAs (Supplementary Figure S2A).

### Identification of IDEmRNAs

Ten significant MREs associated with five circRNAs were identified. To better understand the mechanisms of miRNAs, the target mRNAs were predicted. In total, 3,902 target mRNAs were identified using the TargetScan and miRDB databases. Then, 83 overlapping IDEmRNAs were selected by the intersection of 345 DEmRNAs and 3,902 target mRNAs (Supplementary Figure S2B).

### Construction of the circRNA–miRNA–mRNA network

**FIGURE 3:**
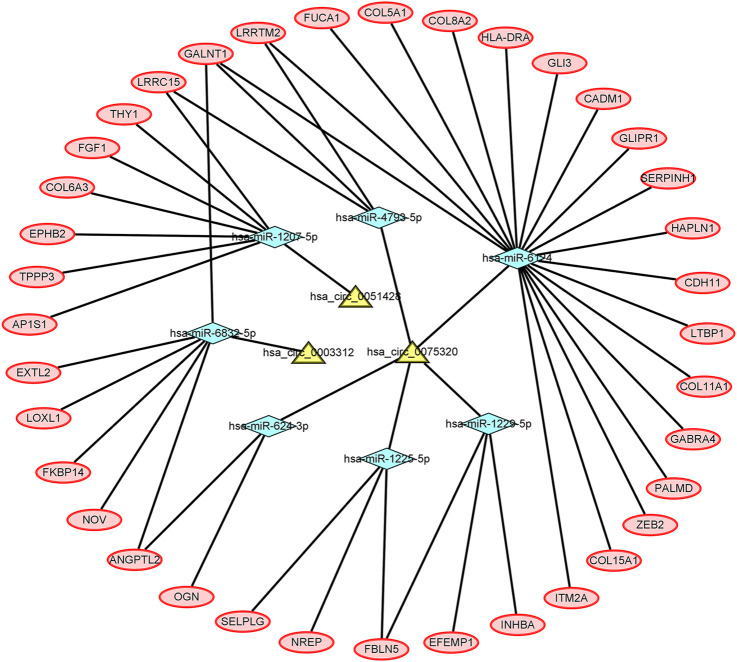
The circRNA–miRNA–mRNA network. The yellow nodes represent the circRNAs, the blue nodes represent the IDEmiRNAs, and the pink nodes represent the IDEmRNAs.

Five DEcircRNAs, 10 IDEmiRNAs, and 83 IDEmRNAs were identified by previous analysis. Based on the above results, a circRNA–miRNA–mRNA network was established, including three circRNAs (hsa_circ_0075320, hsa_circ_0003312, and hsa_circ_0051428), seven miRNAs (hsa-miR-6124, hsa-miR-6832-5p, hsa-miR-1207-5p, hsa-miR-4793-5p, hsa-miR-624-3p, hsa-miR-1225-5p, and hsa-miR-1229-5p), and 37 mRNAs (Figure 3). Heatmaps of circRNAs (Supplementary Figure S3A), miRNAs (Supplementary Figure S3B) and mRNAs (Supplementary Figure S3C) in the network are shown.

### Functional and pathway enrichment analyses

**FIGURE 4:**
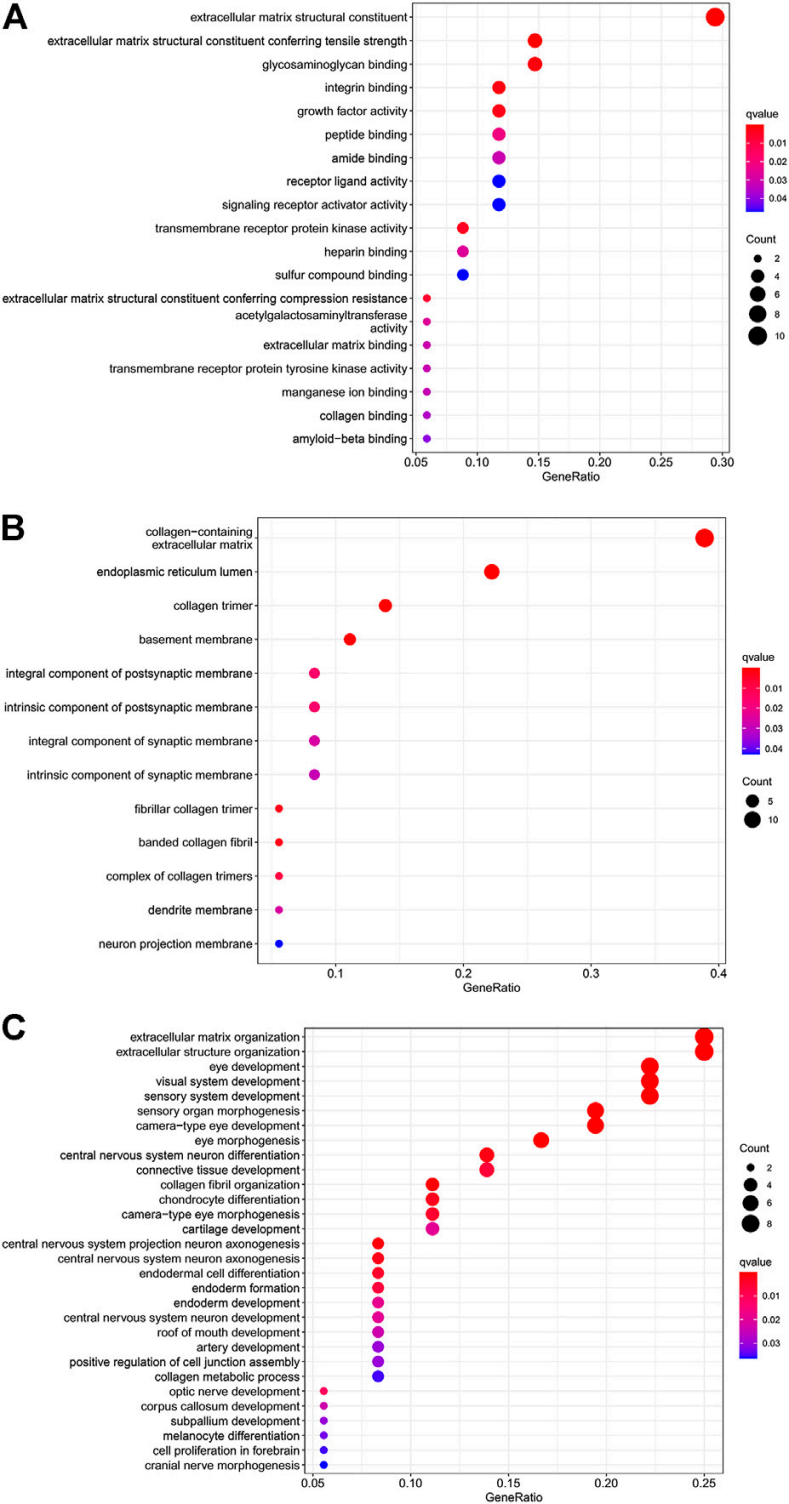
Dot plot of GO function enrichment analysis. **(A)** Molecular function analysis. **(B)** Cellular component analysis. **(C)** Biological process analysis.

**FIGURE 5:**
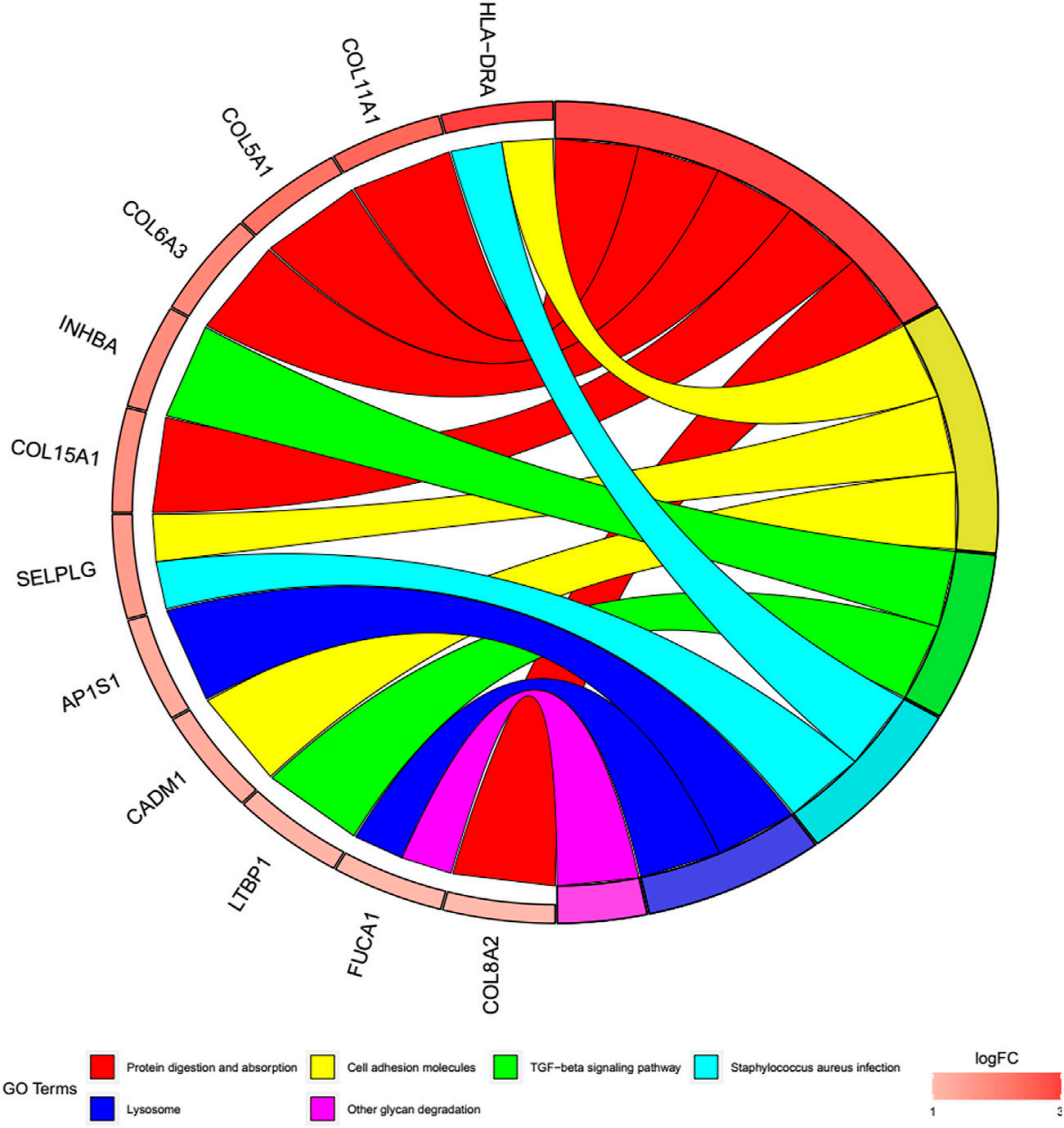
Cohort plot of KEGG pathways analysis shows that twelve genes are associated with six KEGG terms.

To better explore the potential mechanisms of mRNAs in the ceRNA regulatory network, GO enrichment analysis for the terms molecular function (MF), cellular component (CC), and biological process (BP), and KEGG pathway analysis were performed. The top 30 highly enriched GO and KEGG terms are shown in Figures 4, 5. In terms of MF, these genes were primarily clustered in the extracellular matrix structural constituent (*n* = 10), extracellular matrix structural constituent conferring tensile strength (*n* = 5), and glycosaminoglycan binding (*n* = 5) (Figure 4A). In terms of CC, these genes were primarily clustered in the collagen-containing extracellular matrix (*n* = 14) and endoplasmic reticulum lumen (*n* = 8) (Figure 4B). In terms of BP, these genes were primarily clustered in the extracellular matrix (*n* = 9) and extracellular structure (*n* = 9) organization (Figure 4C). According to the results of the KEGG analysis only five pathways, including protein digestion and absorption (*n* = 5), cell adhesion molecules (*n* = 3), transforming growth factor (TGF)-β signaling pathway (*n* = 2), *Staphylococcus aureus* infection (*n* = 2), lysosome, and other glycan degradation (*n* = 1), were enriched (Figure 5).

### Candidate compounds from connectivity map analysis

The mRNAs in the ceRNA network, including the 37 upregulated genes, were uploaded to the CMap website. After searching online, three chemicals (noscapine, diazepam, and TG100-115) with the highest negative enrichment scores were considered potential compounds for the treatment of OA (Supplementary Table S2). The chemical structures of these compounds are shown in Supplementary Figure S4.

### Construction of the protein–protein interaction network and module analysis

**FIGURE 6:**
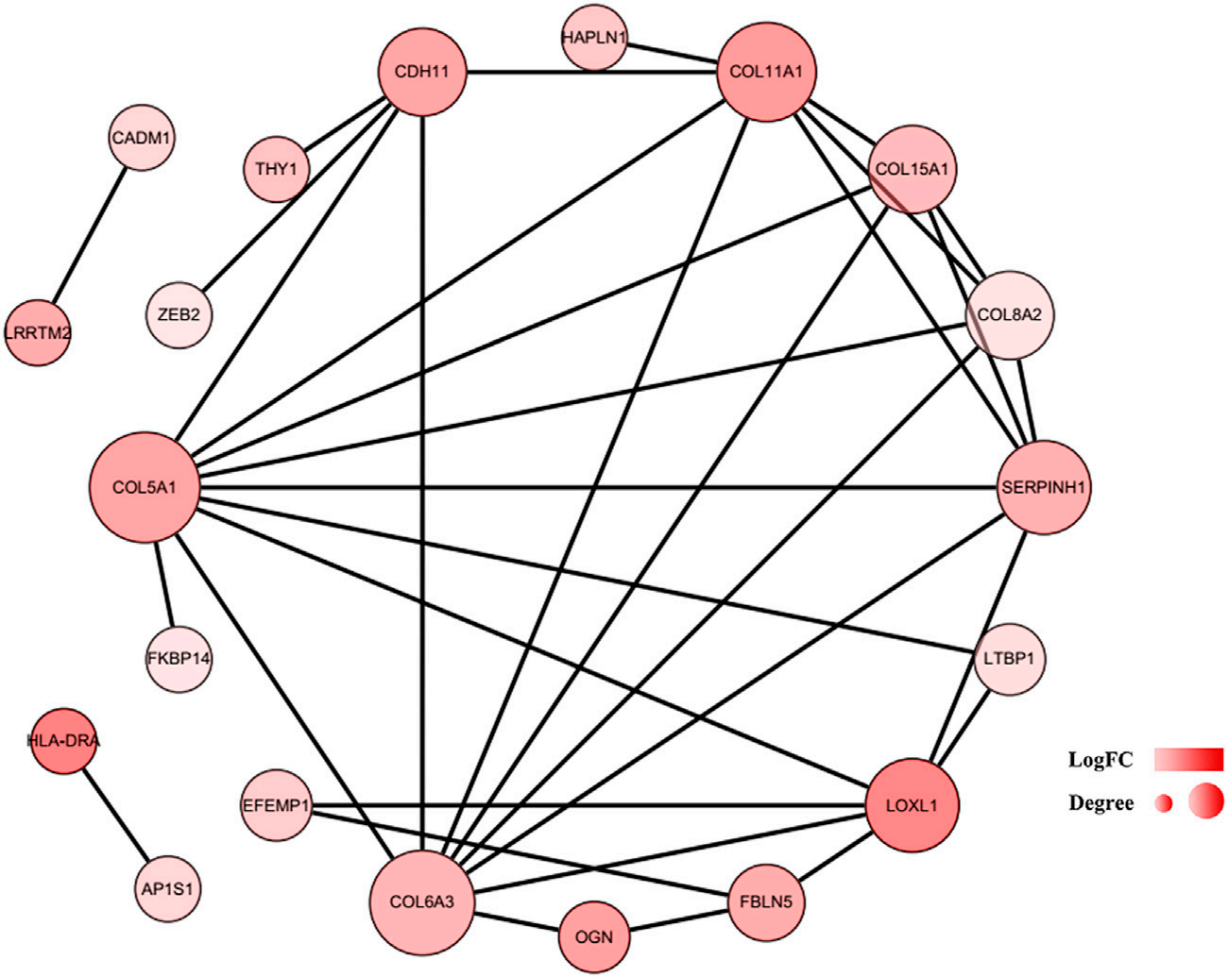
PPI network of 37 mRNAs. The color of a node in the PPI network reflects the log (FC) value of gene expression, and the size of node indicates the number of interacting proteins with the designated protein.

**FIGURE 7:**
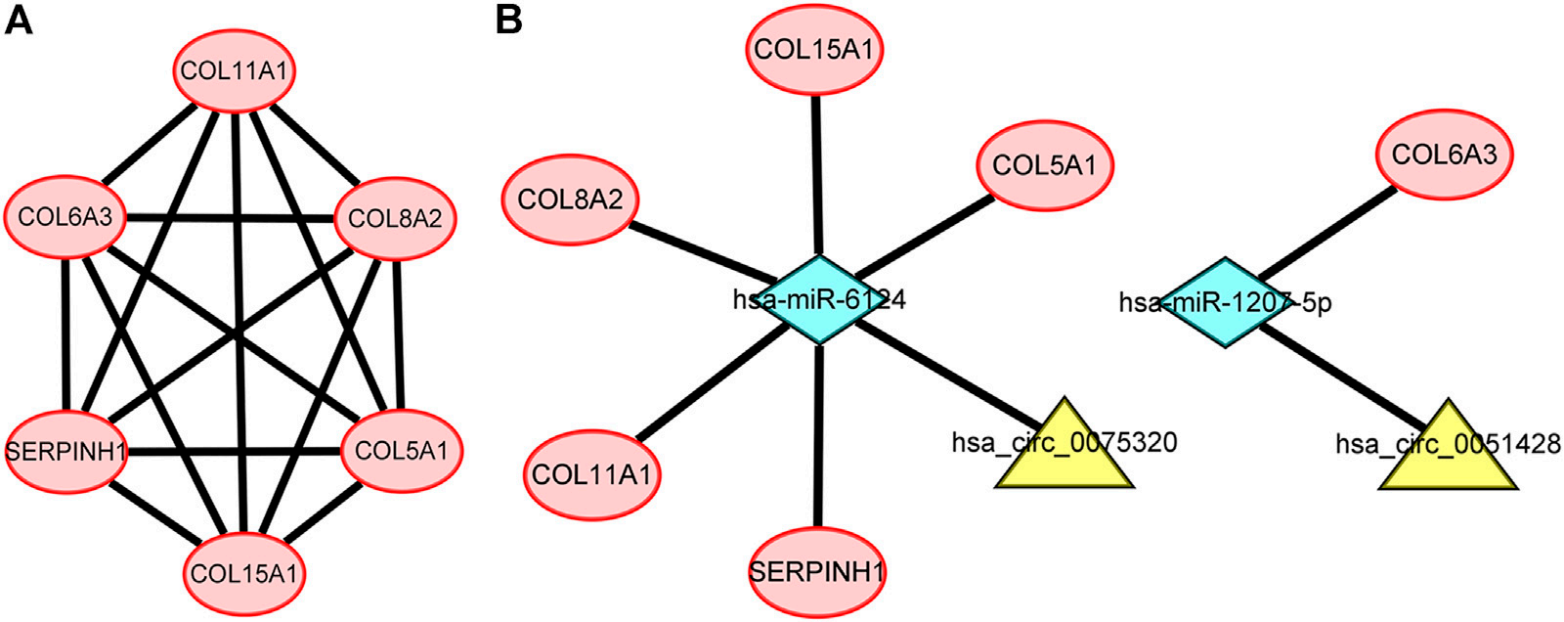
**(A)** PPI network consisting of the six hub genes. **(B)** A subnetwork of circRNAs–miRNAs–hub genes.

To further explore the associations among mRNAs from the ceRNA network, we performed PPI analysis consisting of 18 nodes and 31 edges using the STRING database (Figure 6). Then, the MCODE app in Cytoscape 3.8.0 was used to screen the hub genes. Under the conditions mentioned in above methods, an important module (MCODE score: 5.0) including six nodes and 15 edges was found (Figure 7A), which demonstrates six significant hub genes (serpin family H member 1 [*SERPINH1*], collagen type VIII alpha 2 chain [*COL8A2*], collagen type XV alpha 1 chain [*COL15A1*], collagen type VI alpha 3 chain [*COL6A3*], collagen type V alpha 1 chain [*COL5A1*], and collagen type XI alpha one chain [*COL11A1*]) in OA. The network topological characteristics of six hub genes were showed in Supplementary Table S3. A circRNA-miRNA-hubgene subnetwork was established to identify the associations among the DEcircRNAs, IDEmiRNAs, and hub genes (Figure 7B), and the network included six regulatory axes (hsa_circ_0075320/hsa-miR-6124/*COL11A1*, hsa_circ_0075320/hsa-miR-6124/*SERPINH1*, hsa_circ_0075320/hsa-miR-6124/*COL5A1*, hsa_circ_0075320/hsa-miR-6124/*COL8A2*, hsa_circ_0075320/hsa-miR-6124/*COL15A1*, and hsa_circ_0051428/hsa-miR-1207-5p/*COL6A3*).

### Correlation network analysis for hub genes

**FIGURE 8:**
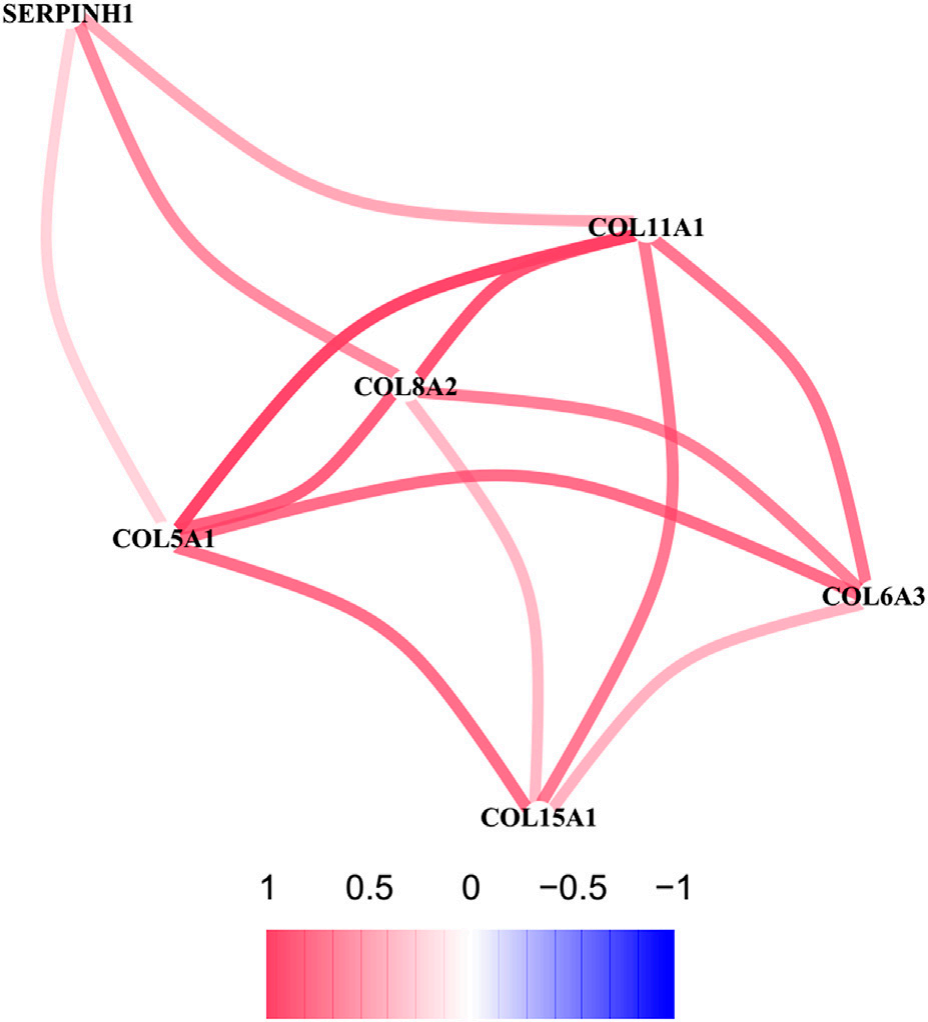
The correlation network of six hub genes. The red line represents a positive correlation between two genes, and the blue represents a negative correlation.

To further explore the sub network identified by MCODE, the correlation network analysis for the six hub genes was performed. The results demonstrated that all six hub genes showed positive correlation, especially among the *COL5A1*, *COL8A2*, and *COL11A1*, whose co-expression was the significant (Figure 8).

### Drug–gene interaction analysis for hub genes

We utilized DGIdb to perform drug–gene interaction analysis for the six key genes identified in the PPI network. Finally, we found that two potential drugs, collagenase *Clostridium histolyticum* and ocriplasmin, may be effective in treating OA because they were found to be closely associated with the four hub genes (*COL15A1*, *COL6A3*, *COL5A1*, *COL11A1*).

## Discussion

OA is a common joints illness that leads to cartilage degeneration, synovial inflammation, and osteophyte formation (Xie and Chen, 2019). However, the pathogenesis of OA is not yet fully understood. Patients with OA usually miss the optimal treatment opportunity due to lack of early diagnostic indicators, leading to a poor prognosis. Therefore, it is important to identify early diagnostic indicators and potential therapeutic targets in patients with OA. In recent years, a bioinformatics-related method has been developed that can be used to predict the potential target genes of various diseases.

CircRNAs are stable ncRNAs that lack a 5′-cap or 3′-tail (Chen et al., 2019). Due to their circular structure, circRNAs identified as biomarkers for diagnosis and prognosis are not affected by RNA exonucleases and become relatively stable(Li et al., 2015; Zhang et al., 2018). Many complex diseases, including non-tumors, are influenced by circRNAs (Xiong et al., 2018; Huo et al., 2021). At present, an increasing number of researchers are focusing on the association between circRNAs and disease occurrence. Some evidence has suggested that circRNAs containing the MRE, RNA binding protein (RBP), and open reading frame (ORF) can combine with miRNAs, thus generally called as miRNA sponges, reducing the cytoplasmic levels of miRNAs and releasing their downstream target mRNAs (He et al., 2017; Bai et al., 2018; Wang et al., 2018). Although one study predicted that hsa_circ_0025119, hsa_circ_0025113, hsa_circ_0009897, and hsa_circ_0002447 exert significant effects on OA, the specific functions of circRNAs remain unknown (Wang et al., 2021). Therefore, to determine whether circRNAs function as ceRNAs in OA, we constructed a circRNA–miRNA–mRNA regulatory network based on biological forecasts, a PPI network based on STRING, and a sub network based on circRNAs, miRNAs, and hub genes.

In this study, five DEcircRNAs, 89 DEmiRNAs, and 345 DEmRNAs were first obtained from the GSE175959, GSE105027, and GSE169077 datasets, respectively. To date, these five DE circRNAs (hsa_circ_0004662, hsa_circ_0051428, hsa_circ_0003312, hsa_circ_0008590, and hsa_circ_0075320) have not been reported in previously published articles. circRNAs are ceRNAs that alter the gene expression by binding to miRNAs (Zhang et al., 2017; Zhong et al., 2018; Kim et al., 2021). To investigate whether the five circRNAs have a ceRNA role in OA, the circBase and CSCD databases were used to predict their MREs. To further explore the mechanisms of miRNAs and mRNAs of circRNAs downstream in OA, the miRDB and TargetScan databases were utilized to forecast target genes. Finally, a circRNAs-miRNA-mRNAs regulatory network was established based on three circRNAs (hsa_circ_0075320, hsa_circ_0003312, and hsa_circ_0051428), seven miRNAs (hsa-miR-6124, hsa-miR-6832-5p, hsa-miR-1207-5p, hsa-miR-4793-5p, hsa-miR-624-3p, hsa-miR-1225-5p, and hsa-miR-1229-5p), and 37 mRNAs.

We then conducted enrichment analysis based on the mRNAs in this network. The analysis demonstrated that the structural constituents of the extracellular matrix (ECM), such as collagen and glycosaminoglycan, are closely associated with the occurrence and progression of OA. Furthermore, type II collagen in cartilage ECM is disintegrated via increased expression of cartilage-degrading enzymes, ultimately resulting in the degeneration of articular cartilage (Glasson et al., 2005; Stanton et al., 2005). Aberrant crosslinking of collagen fibrils within the joint may result in OA (Hu et al., 2006; Wang et al., 2008). In this study, pathways detected by KEGG analysis were mainly related to protein digestion and absorption, cell adhesion molecules, TGF-β signaling pathway, *S. aureus* infection, lysosomes, and other glycan degradation. Cartilage damage is a major issue in OA. In recent years, there is moderate evidence showing that growth factors such as TGF-β have great potential in cartilage repair (Blaney Davidson et al., 2007; Wang et al., 2020). TGF-β can prevent the occurrence of osteoarthritis by maintaining the homeostasis of articular cartilage, and TGF-β supplementation can also enhance cartilage repair, which is a potential treatment method (Wang et al., 2020). According to the relevant research, some cell adhesion molecules can be used as predictors of OA (Schett et al., 2009). Therefore, the cell adhesion molecules are also closely relevant to the disease process for OA.

To further determine the key circRNAs involved in the ceRNA network, a PPI network was constructed, and six hub genes (*SERPINH1*, *COL5A1*, *COL15A1*, *COL6A3*, *COL8A2*, and *COL11A1*) were identified using the MCODE app. Bioinformatics analysis revealed that these hub genes are likely to play significant roles in the pathogenesis of OA. In previous studies, *SERPINH1*, a collagen-specific molecular chaperone critical for type I and type III collagen maturation,(Taguchi and Razzaque, 2007) was shown to be associated with OA via proteomic analysis (Tsolis et al., 2015). Consistent with our results, aberrant expression of *COL5A1* was previously shown to be a target gene in synovial fibrosis therapy (Remst et al., 2014). *COL15A1*, whose expression was confirmed to be increased in OA, can encode a protein with significant expression in newly formed blood vessels, revealing a probable function in angiogenesis (Karlsson et al., 2010). *COL6A3* is associated with cartilage degeneration, as verified by quantitative PCR analysis (Chou et al., 2013). *COL8A2*, a collagen-related gene, is more abundantly expressed in OA, but the exact mechanism is not fully understood (Karlsson et al., 2010). A meta-analysis demonstrated that common missense variants in *COL11A1* are associated with OA (Styrkarsdottir et al., 2018). *COL11A1* contributes to the development of OA due to the increased degradation of articular cartilage type II collagen caused by the type XI collagen mutation (Rodriguez et al., 2004). However, the mechanisms of action of the identified hub genes in OA need to be further explored.

Although the main functions of the six key genes have been described, their connections with circRNAs are yet to be explored. In this study, six circRNA–miRNA–hubgene subnetwork regulatory axes (hsa_circ_0075320/hsa-miR-6124/*COL11A1*, hsa_circ_0075320/hsa-miR-6124/*SERPINH1*, hsa_circ_0075320/hsa-miR-6124/*COL5A1*, hsa_circ_0075320/hsa-miR-6124/*COL8A2*, hsa_circ_0075320/hsa-miR-6124/*COL15A1*, hsa_circ_0051428/hsa-miR-1207-5p/*COL6A3*) were identified, suggesting competitive regulatory relationships between hsa_circ_0075320/hsa_circ_0051428 and the six hub genes. Nevertheless, considering that these outcomes were derived from bioinformatics analysis, additional experiments must be performed to validate the possible effects of these six axes.

Currently, pharmacotherapy is vital in treating OA, with most patients requiring short-term or long-term drug therapy. CMap analysis was performed to identify potential therapeutic agents. CMap analysis identified three compounds (noscapine, diazepam, and TG100-115) as candidate drugs, facilitating the exploration of their corresponding targets. Although noscapine is an alkaloid derived from opioids, it has been clinically used as an antitussive agent without any addictive or euphoric effects. The association between noscapine and OA has not yet been reported, but noscapine is suggested to act as an anti-inflammatory agent that remarkably decreases the levels of proinflammatory factors and shows antioxidant effects by inhibiting nitric oxide and reducing reactive oxygen species levels (Rahmanian-Devin et al., 2021). These therapeutic effects, combined with its low systemic toxicity, make noscapine a potential candidate for the treatment of various inflammatory diseases, including OA. Thomas et al. (1991) have demonstrated the effectiveness of diazepam in OA treatment. However, the long-term efficacy of diazepam in OA treatment requires further investigation and verification. Doukas et al. confirmed that TG100-115, a selective phosphatidylinositol 3-kinase inhibitor, can reduce infarct development and preserve myocardial function by effectively inhibiting edema and inflammation (Doukas et al., 2006). Although the link between TG100-115 and OA remains unclear, we believe that the occurrence and progression of OA can be controlled by effectively inhibiting edema and inflammation.

In addition to the CMap analysis described above, hub genes were found to be significant in OA. Hence, we conducted a drug–gene interaction analysis using the DGIdb online database and identified two related drugs, collagenase *Clostridium histolyticum* and ocriplasmin, for OA treatment. At present, ocriplasmin is mainly used to treat some ophthalmic diseases (Pirani et al., 2019), while collagenase *Clostridium histolyticum* is primarily used to treat Dupuytren’s contracture or Peyronie’s disease (Abdel Raheem et al., 2018; Fletcher et al., 2019). Nevertheless, whether such medications have satisfactory effects on OA needs to be further investigated.

In our study, six circRNA–miRNA–hubgene axes were ultimately identified, revealing competitive interactions between two circRNAs and six hub genes in OA. In the future, more experiments are needed to validate this probable ceRNA mechanism in OA.

However, there are several limitations to this research. Firstly, this study was done primarily using bioinformatics analysis; therefore, further experimental validation is needed. Secondly, the sample size was not large. Finally, further clinical studies should be performed to explore the correlation between these findings in this study and clinical practice.

## Conclusion

In summary, a circRNA–miRNA–mRNA regulatory network was established, including three important circRNAs (hsa_circ_0075320, hsa_circ_0003312, and hsa_circ_0051428) *via* comprehensive bioinformatics analyses. We then constructed a circRNA–miRNA–hubgene regulatory subnetwork involving two vitally important circRNAs, hsa_circ_0075320, hsa_circ_0051428. Furthermore, three bioactive compounds, noscapine, diazepam, and TG100-115, according to the CMap analysis, and two related drugs, collagenase *Clostridium histolyticum* and ocriplasmin, according to the DGIdb database, were identified as potential treatment options for OA. This study reveals a unique perspective of the regulatory mechanisms and possible drugs for OA treatment from the ceRNA network view.

## Data Availability

Publicly available datasets were analyzed in this study. The names of the repository/repositories and accession number(s) can be found in the article/Supplementary Material.

## Supplementary material

SUPPLEMENTAY FIGURE S1 Heatmaps of the differentially expressed circRNAs (DEcircRNAs), microRNAs (DEcircRNAs), and mRNAs (DEmRNAs).**(A)** Heatmap of the DEcircRNAs in the GSE175959 dataset. **(B)** Heatmap of the DEmiRNAs in the GSE105027 dataset. **(C)** Heatmap of the DEmRNAs in the GSE105027 dataset.

SUPPLEMENTAY FIGURE S2
**(A)** Venn diagram of the 89 differentially expressed miRNAs (DEmiRNAs) obtained from GSE105027 and their overlap with the MREs. **(B)** Venn diagram of the 345 differentially expressed mRNAs (DEmRNAs) obtained from GSE169077 and their overlap with miRNA target genes.

SUPPLEMENTAY FIGURE S3
**(A)** Heatmap of the three circular RNAs (circRNAs) in the competing endogenous RNA (ceRNA) network. **(B)** Heatmap of the seven miRNAs in the ceRNA network. **(C)** Heatmap of the 37 mRNAs in the ceRNA network.

SUPPLEMENTAY FIGURE S4 Chemical structures of the three compounds found by the CMap analysis. **(A)** Noscapine. **(B)** Diazepam. **(C)** TG100-115.

## References

[B1] Abdel Raheem A. Johnson M. Ralph D. Garaffa G. (2018). Collagenase clostridium histolyticum: A novel medical treatment for Peyronie's disease. Minerva Urol. Nefrol. 70 (4), 380–385. 10.23736/s0393-2249.18.03118-1 29761688

[B2] Agarwal V. Bell G. W. Nam J. W. Bartel D. P. (2015). Predicting effective microRNA target sites in mammalian mRNAs. Elife 4. 10.7554/eLife.05005 PMC4532895 26267216

[B3] Bader G. D. Hogue C. W. (2003). An automated method for finding molecular complexes in large protein interaction networks. BMC Bioinforma. 4, 2. 10.1186/1471-2105-4-2 PMC149346 12525261

[B4] Bai N. Peng E. Qiu X. Lyu N. Zhang Z. Tao Y. (2018). circFBLIM1 act as a ceRNA to promote hepatocellular cancer progression by sponging miR-346. J. Exp. Clin. Cancer Res. 37 (1), 172. 10.1186/s13046-018-0838-8 30053867 PMC6062991

[B5] Barrett T. Wilhite S. E. Ledoux P. Evangelista C. Kim I. F. Tomashevsky M. (2013). NCBI GEO: Archive for functional genomics data sets--update. Nucleic Acids Res. 41, D991–D995. Database issue). 10.1093/nar/gks1193 23193258 PMC3531084

[B6] Blaney Davidson E. N. van der Kraan P. M. van den Berg W. B. (2007). TGF-beta and osteoarthritis. Osteoarthr. Cartil. 15 (6), 597–604. 10.1016/j.joca.2007.02.005 17391995

[B7] Chen B. J. Huang S. Janitz M. (2019). Changes in circular RNA expression patterns during human foetal brain development. Genomics 111 (4), 753–758. 10.1016/j.ygeno.2018.04.015 29709512

[B8] Chou C. H. Lee C. H. Lu L. S. Song I. W. Chuang H. P. Kuo S. Y. (2013). Direct assessment of articular cartilage and underlying subchondral bone reveals a progressive gene expression change in human osteoarthritic knees. Osteoarthr. Cartil. 21 (3), 450–461. 10.1016/j.joca.2012.11.016 PMC3593157 23220557

[B9] Cross M. Smith E. Hoy D. Nolte S. Ackerman I. Fransen M. (2014). The global burden of hip and knee osteoarthritis: Estimates from the global burden of disease 2010 study. Ann. Rheum. Dis. 73 (7), 1323–1330. 10.1136/annrheumdis-2013-204763 24553908

[B10] Doukas J. Wrasidlo W. Noronha G. Dneprovskaia E. Fine R. Weis S. (2006). Phosphoinositide 3-kinase gamma/delta inhibition limits infarct size after myocardial ischemia/reperfusion injury. Proc. Natl. Acad. Sci. U. S. A. 103 (52), 19866–19871. 10.1073/pnas.0606956103 17172449 PMC1702529

[B11] Fletcher J. Tan E. S. L. Thomas M. Taylor F. Elliott D. Bindra R. (2019). Collagenase injections for Dupuytren's contracture: Prospective cohort study in a public health setting. ANZ J. Surg. 89 (5), 573–577. 10.1111/ans.14988 30685881

[B12] Glasson S. S. Askew R. Sheppard B. Carito B. Blanchet T. Ma H. L. (2005). Deletion of active ADAMTS5 prevents cartilage degradation in a murine model of osteoarthritis. Nature 434 (7033), 644–648. 10.1038/nature03369 15800624

[B13] Glažar P. Papavasileiou P. Rajewsky N. (2014). circBase: a database for circular RNAs. Rna 20 (11), 1666–1670. 10.1261/rna.043687.113 25234927 PMC4201819

[B14] Griffith M. Griffith O. L. Coffman A. C. Weible J. V. McMichael J. F. Spies N. C. (2013). DGIdb: Mining the druggable genome. Nat. Methods 10 (12), 1209–1210. 10.1038/nmeth.2689 24122041 PMC3851581

[B15] He R. Liu P. Xie X. Zhou Y. Liao Q. Xiong W. (2017). circGFRA1 and GFRA1 act as ceRNAs in triple negative breast cancer by regulating miR-34a. J. Exp. Clin. Cancer Res. 36 (1), 145. 10.1186/s13046-017-0614-1 29037220 PMC5644184

[B16] Hu K. Xu L. Cao L. Flahiff C. M. Brussiau J. Ho K. (2006). Pathogenesis of osteoarthritis-like changes in the joints of mice deficient in type IX collagen. Arthritis Rheum. 54 (9), 2891–2900. 10.1002/art.22040 16947423

[B17] Huo Z. Li H. Tian L. Li J. Zhang K. Li Z. (2021). Construction of a potentially functional circRNA-miRNA-mRNA network in intervertebral disc degeneration by bioinformatics analysis. Biomed. Res. Int. 2021, 8352683. 10.1155/2021/8352683 34395625 PMC8357516

[B18] Kanehisa M. Goto S. (2000). Kegg: Kyoto encyclopedia of genes and genomes. Nucleic Acids Res. 28 (1), 27–30. 10.1093/nar/28.1.27 10592173 PMC102409

[B19] Karlsson C. Dehne T. Lindahl A. Brittberg M. Pruss A. Sittinger M. (2010). Genome-wide expression profiling reveals new candidate genes associated with osteoarthritis. Osteoarthr. Cartil. 18 (4), 581–592. 10.1016/j.joca.2009.12.002 20060954

[B20] Kiadaliri A. A. Lohmander L. S. Moradi-Lakeh M. Petersson I. F. Englund M. (2018). High and rising burden of hip and knee osteoarthritis in the Nordic region, 1990-2015. Acta Orthop. 89 (2), 177–183. 10.1080/17453674.2017.1404791 29160139 PMC5901515

[B21] Kim E. Kim Y. K. Lee S. V. (2021). Emerging functions of circular RNA in aging. Trends Genet. 37 (9), 819–829. 10.1016/j.tig.2021.04.014 34016449

[B22] Kristensen L. S. Hansen T. B. Venø M. T. Kjems J. (2018). Circular RNAs in cancer: Opportunities and challenges in the field. Oncogene 37 (5), 555–565. 10.1038/onc.2017.361 28991235 PMC5799710

[B23] Lamb J. Crawford E. D. Peck D. Modell J. W. Blat I. C. Wrobel M. J. (2006). The connectivity map: Using gene-expression signatures to connect small molecules, genes, and disease. Science 313 (5795), 1929–1935. 10.1126/science.1132939 17008526

[B24] Lespasio M. J. Piuzzi N. S. Husni M. E. Muschler G. F. Guarino A. Mont M. A. (2017). Knee osteoarthritis: A primer. Perm. J. 21, 16–183. 10.7812/tpp/16-183 PMC5638628 29035179

[B25] Li J. Yang J. Zhou P. Le Y. Zhou C. Wang S. (2015a). Circular RNAs in cancer: Novel insights into origins, properties, functions and implications. Am. J. Cancer Res. 5 (2), 472–480. 25973291 PMC4396047

[B26] Li P. Chen S. Chen H. Mo X. Li T. Shao Y. (2015b). Using circular RNA as a novel type of biomarker in the screening of gastric cancer. Clin. Chim. Acta. 444, 132–136. 10.1016/j.cca.2015.02.018 25689795

[B27] Petkovic S. Müller S. (2015). RNA circularization strategies *in vivo* and *in vitro* . Nucleic Acids Res. 43 (4), 2454–2465. 10.1093/nar/gkv045 25662225 PMC4344496

[B28] Pirani V. Pelliccioni P. Cesari C. Carrozzi G. Cavallero E. Mariotti C. (2019). Flare changes after intravitreal injection of ocriplasmin in symptomatic vitreomacular traction syndrome. Jpn. J. Ophthalmol. 63 (3), 255–261. 10.1007/s10384-019-00660-z 30805734

[B29] Qi X. Zhang D. H. Wu N. Xiao J. H. Wang X. Ma W. (2015). ceRNA in cancer: possible functions and clinical implications. J. Med. Genet. 52 (10), 710–718. 10.1136/jmedgenet-2015-103334 26358722

[B30] Qu S. Yang X. Li X. Wang J. Gao Y. Shang R. (2015). Circular RNA: A new star of noncoding RNAs. Cancer Lett. 365 (2), 141–148. 10.1016/j.canlet.2015.06.003 26052092

[B31] Rahmanian-Devin P. Baradaran Rahimi V. Jaafari M. R. Golmohammadzadeh S. Sanei-Far Z. Askari V. R. (2021). Noscapine, an emerging medication for different diseases: A mechanistic review. Evid. Based. Complement. Altern. Med. 2021, 8402517. 10.1155/2021/8402517 PMC8648453 34880922

[B32] Remst D. F. Blom A. B. Vitters E. L. Bank R. A. van den Berg W. B. Blaney Davidson E. N. (2014). Gene expression analysis of murine and human osteoarthritis synovium reveals elevation of transforming growth factor β-responsive genes in osteoarthritis-related fibrosis. Arthritis Rheumatol. 66 (3), 647–656. 10.1002/art.38266 24574225

[B33] Ritchie M. E. Phipson B. Wu D. Hu Y. Law C. W. Shi W. (2015). Limma powers differential expression analyses for RNA-sequencing and microarray studies. Nucleic Acids Res. 43 (7), e47. 10.1093/nar/gkv007 25605792 PMC4402510

[B34] Rodriguez R. R. Seegmiller R. E. Stark M. R. Bridgewater L. C. (2004). A type XI collagen mutation leads to increased degradation of type II collagen in articular cartilage. Osteoarthr. Cartil. 12 (4), 314–320. 10.1016/j.joca.2003.12.002 15023383

[B35] Salzman J. Chen R. E. Olsen M. N. Wang P. L. Brown P. O. (2013). Cell-type specific features of circular RNA expression. PLoS Genet. 9 (9), e1003777. 10.1371/journal.pgen.1003777 24039610 PMC3764148

[B36] Schett G. Kiechl S. Bonora E. Zwerina J. Mayr A. Axmann R. (2009). Vascular cell adhesion molecule 1 as a predictor of severe osteoarthritis of the hip and knee joints. Arthritis Rheum. 60 (8), 2381–2389. 10.1002/art.24757 19644856

[B37] Stanton H. Rogerson F. M. East C. J. Golub S. B. Lawlor K. E. Meeker C. T. (2005). ADAMTS5 is the major aggrecanase in mouse cartilage *in vivo* and *in vitro* . Nature 434 (7033), 648–652. 10.1038/nature03417 15800625

[B38] Styrkarsdottir U. Lund S. H. Thorleifsson G. Zink F. Stefansson O. A. Sigurdsson J. K. (2018). Meta-analysis of Icelandic and UK data sets identifies missense variants in SMO, IL11, COL11A1 and 13 more new loci associated with osteoarthritis. Nat. Genet. 50 (12), 1681–1687. 10.1038/s41588-018-0247-0 30374069

[B39] Su G. Morris J. H. Demchak B. Bader G. D. (2014). Biological network exploration with Cytoscape 3. Curr. Protoc. Bioinforma. 47, 811–1324. 10.1002/0471250953.bi0813s47 PMC4174321 25199793

[B40] Szklarczyk D. Morris J. H. Cook H. Kuhn M. Wyder S. Simonovic M. (2017). The STRING database in 2017: Quality-controlled protein-protein association networks, made broadly accessible. Nucleic Acids Res. 45 (D1), D362–d368. 10.1093/nar/gkw937 27924014 PMC5210637

[B41] Taguchi T. Razzaque M. S. (2007). The collagen-specific molecular chaperone HSP47: Is there a role in fibrosis? Trends Mol. Med. 13 (2), 45–53. 10.1016/j.molmed.2006.12.001 17169614

[B42] Tang X. Wang S. Zhan S. Niu J. Tao K. Zhang Y. (2016). The prevalence of symptomatic knee osteoarthritis in China: Results from the China health and retirement longitudinal study. Arthritis Rheumatol. 68 (3), 648–653. 10.1002/art.39465 26474054

[B43] Thomas M. Eriksson S. V. Lundeberg T. (1991). A comparative study of diazepam and acupuncture in patients with osteoarthritis pain: A placebo controlled study. Am. J. Chin. Med. 19 (2), 95–100. 10.1142/s0192415x91000156 1816730

[B44] Tsolis K. C. Bei E. S. Papathanasiou I. Kostopoulou F. Gkretsi V. Kalantzaki K. (2015). Comparative proteomic analysis of hypertrophic chondrocytes in osteoarthritis. Clin. Proteomics 12 (1), 12. 10.1186/s12014-015-9085-6 25945082 PMC4415313

[B45] Vidal A. F. Sandoval G. T. Magalhães L. Santos S. E. Ribeiro-dos-Santos Â. (2016). Circular RNAs as a new field in gene regulation and their implications in translational research. Epigenomics 8 (4), 551–562. 10.2217/epi.16.3 27035397

[B46] Wang B. Zhong J. L. Xu X. H. Wu B. Shang J. Jiang N. (2021). Gene expression profiling analysis to identify key genes and underlying mechanisms in meniscus of osteoarthritis patients. Comb. Chem. High. Throughput Screen. 24 (8), 1151–1167. 10.2174/1386207323666200902140656 32881662

[B47] Wang C. J. Iida K. Egusa H. Hokugo A. Jewett A. Nishimura I. (2008). Trabecular bone deterioration in col9a1+/- mice associated with enlarged osteoclasts adhered to collagen IX-deficient bone. J. Bone Min. Res. 23 (6), 837–849. 10.1359/jbmr.080214 PMC2677084 18251701

[B48] Wang C. Shen J. Ying J. Xiao D. O'Keefe R. J. (2020). FoxO1 is a crucial mediator of TGF-β/TAK1 signaling and protects against osteoarthritis by maintaining articular cartilage homeostasis. Proc. Natl. Acad. Sci. U. S. A. 117 (48), 30488–30497. 10.1073/pnas.2017056117 33199631 PMC7720227

[B49] Wang W. L. Yang Z. Zhang Y. J. Lu P. Ni Y. K. Sun C. F. (2018). Competing endogenous RNA analysis reveals the regulatory potency of circRNA_036186 in HNSCC. Int. J. Oncol. 53 (4), 1529–1543. 10.3892/ijo.2018.4499 30066847 PMC6086620

[B50] Wei H. Pan L. Tao D. Li R. (2018). Circular RNA circZFR contributes to papillary thyroid cancer cell proliferation and invasion by sponging miR-1261 and facilitating C8orf4 expression. Biochem. Biophys. Res. Commun. 503 (1), 56–61. 10.1016/j.bbrc.2018.05.174 29842886

[B51] Wong N. Wang X. (2015). miRDB: an online resource for microRNA target prediction and functional annotations. Nucleic Acids Res. 43, D146–D152. 10.1093/nar/gku1104 25378301 PMC4383922

[B52] Xia S. Feng J. Chen K. Ma Y. Gong J. Cai F. (2018). Cscd: A database for cancer-specific circular RNAs. Nucleic Acids Res. 46 (D1), D925–d929. 10.1093/nar/gkx863 29036403 PMC5753219

[B53] Xie C. Chen Q. (2019). Adipokines: New therapeutic target for osteoarthritis? Curr. Rheumatol. Rep. 21 (12), 71. 10.1007/s11926-019-0868-z 31813080 PMC7291783

[B54] Xiong D. D. Dang Y. W. Lin P. Wen D. Y. He R. Q. Luo D. Z. (2018). A circRNA-miRNA-mRNA network identification for exploring underlying pathogenesis and therapy strategy of hepatocellular carcinoma. J. Transl. Med. 16 (1), 220. 10.1186/s12967-018-1593-5 30092792 PMC6085698

[B55] Yu G. Wang L. G. Han Y. He Q. Y. (2012). clusterProfiler: an R package for comparing biological themes among gene clusters. Omics 16 (5), 284–287. 10.1089/omi.2011.0118 22455463 PMC3339379

[B56] Zhang H. D. Jiang L. H. Sun D. W. Hou J. C. Ji Z. L. (2018). CircRNA: A novel type of biomarker for cancer. Breast Cancer 25 (1), 1–7. 10.1007/s12282-017-0793-9 28721656

[B57] Zhang Y. Liang W. Zhang P. Chen J. Qian H. Zhang X. (2017). Circular RNAs: Emerging cancer biomarkers and targets. J. Exp. Clin. Cancer Res. 36 (1), 152. 10.1186/s13046-017-0624-z 29096676 PMC5667461

[B58] Zhong Y. Du Y. Yang X. Mo Y. Fan C. Xiong F. (2018). Circular RNAs function as ceRNAs to regulate and control human cancer progression. Mol. Cancer 17 (1), 79. 10.1186/s12943-018-0827-8 29626935 PMC5889847

