# Supplementary material for: Identification of the circRNA–miRNA–mRNA regulatory network in osteoarthritis using bioinformatics analysis
Source: Front Genet. 2022 Sep 16;13:994163. doi: 10.3389/fgene.2022.994163 (PMC9523487; doi:10.3389/fgene.2022.994163)
Supplement: Supplementary file 1 [file Table1.DOCX]

**Table | S1.** Essential information of the 5 DEcircRNAs

| circRNA ID | Position | Strand | Best transcript | Gene symbol | Regulation |
| --- | --- | --- | --- | --- | --- |
| hsa_circ_0004662 | chr6:160103505-160109274 | - | NM_001024465 | SOD2 | Up |
| hsa_circ_0051428 | chr19:45525310-45528995 | + | NM_006509 | RELB | Up |
| hsa_circ_0003312 | chr19:45528586-45532250 | + | NM_006509 | RELB | Up |
| hsa_circ_0008590 | chr19:45528586-45528995 | + | NM_006509 | RELB | Up |
| hsa_circ_0075320 | chr5:179249957-179260782 | + | NM_001142298 | SQSTM1 | Up |

DEcircRNAs, differentially expressed circRNAs.
