# Supplementary figures and images for: Identification of the circRNA–miRNA–mRNA regulatory network in osteoarthritis using bioinformatics analysis
Source: Front Genet. 2022 Sep 16;13:994163. doi: 10.3389/fgene.2022.994163 (PMC9523487; doi:10.3389/fgene.2022.994163)

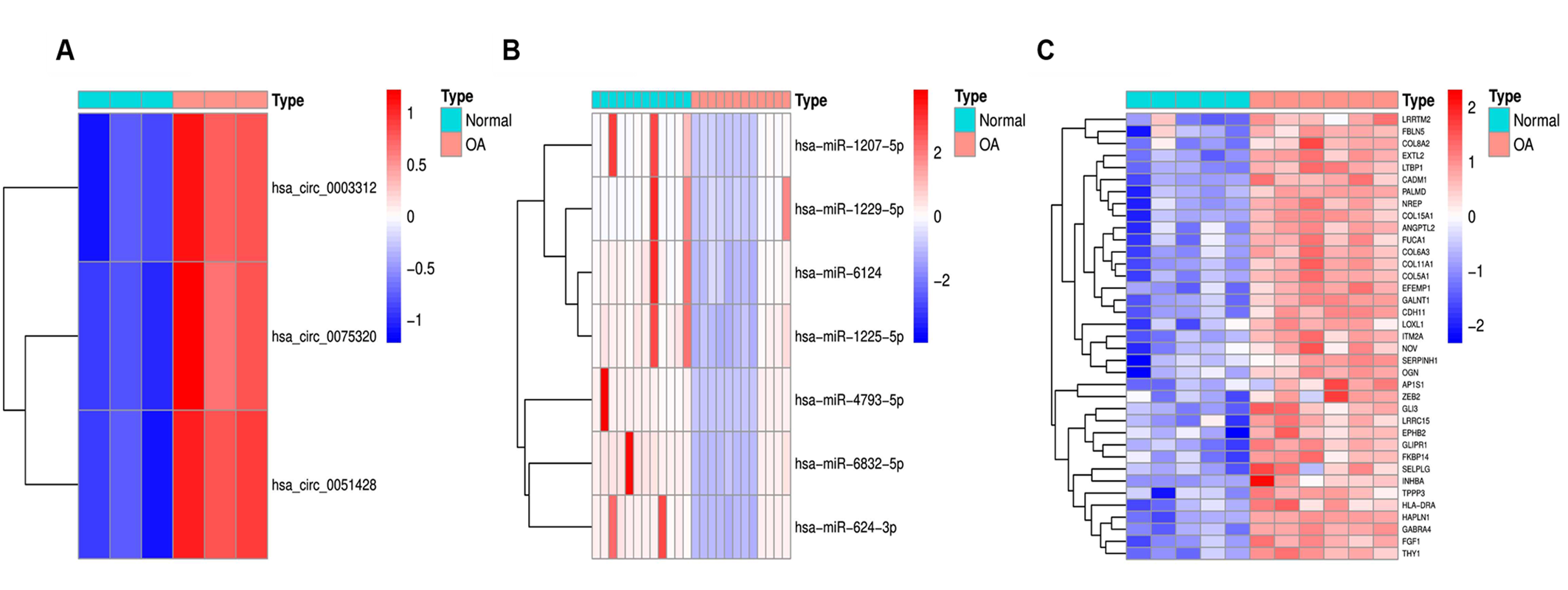

Supplement: Supplementary file 2 [file Image3.TIF]

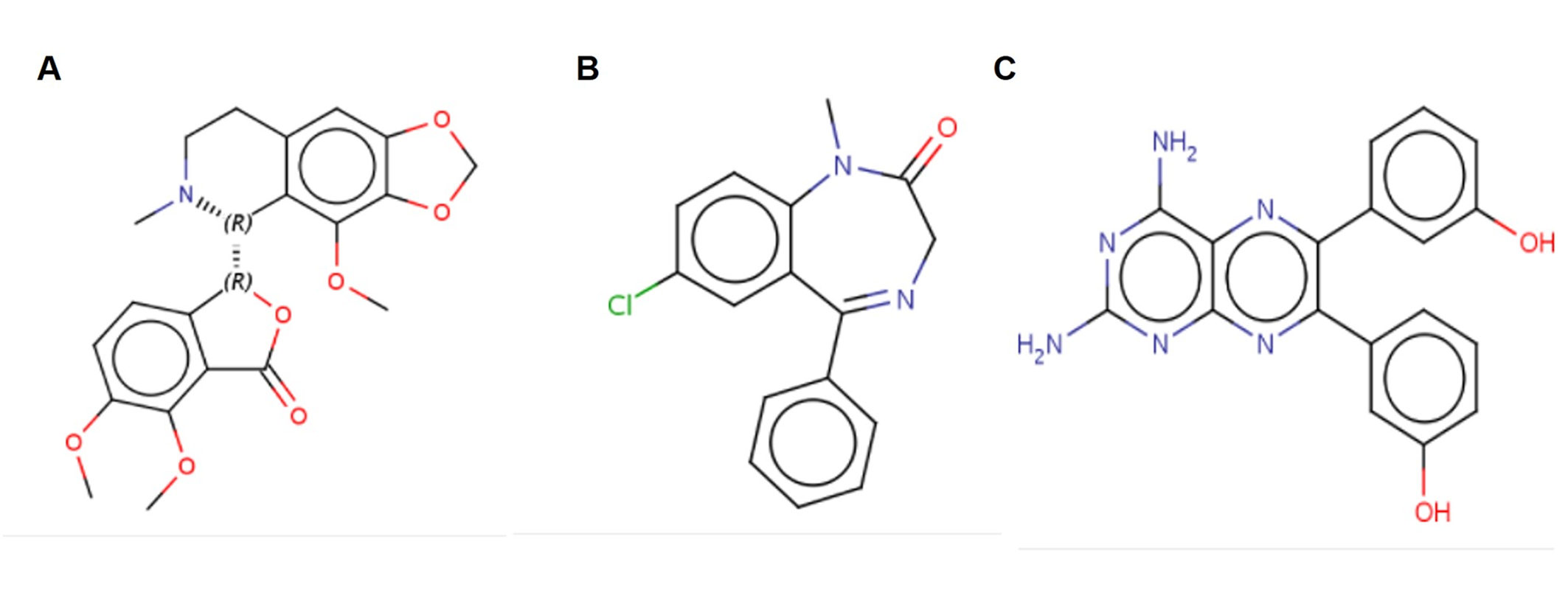

Supplement: Supplementary file 3 [file Image4.TIF]

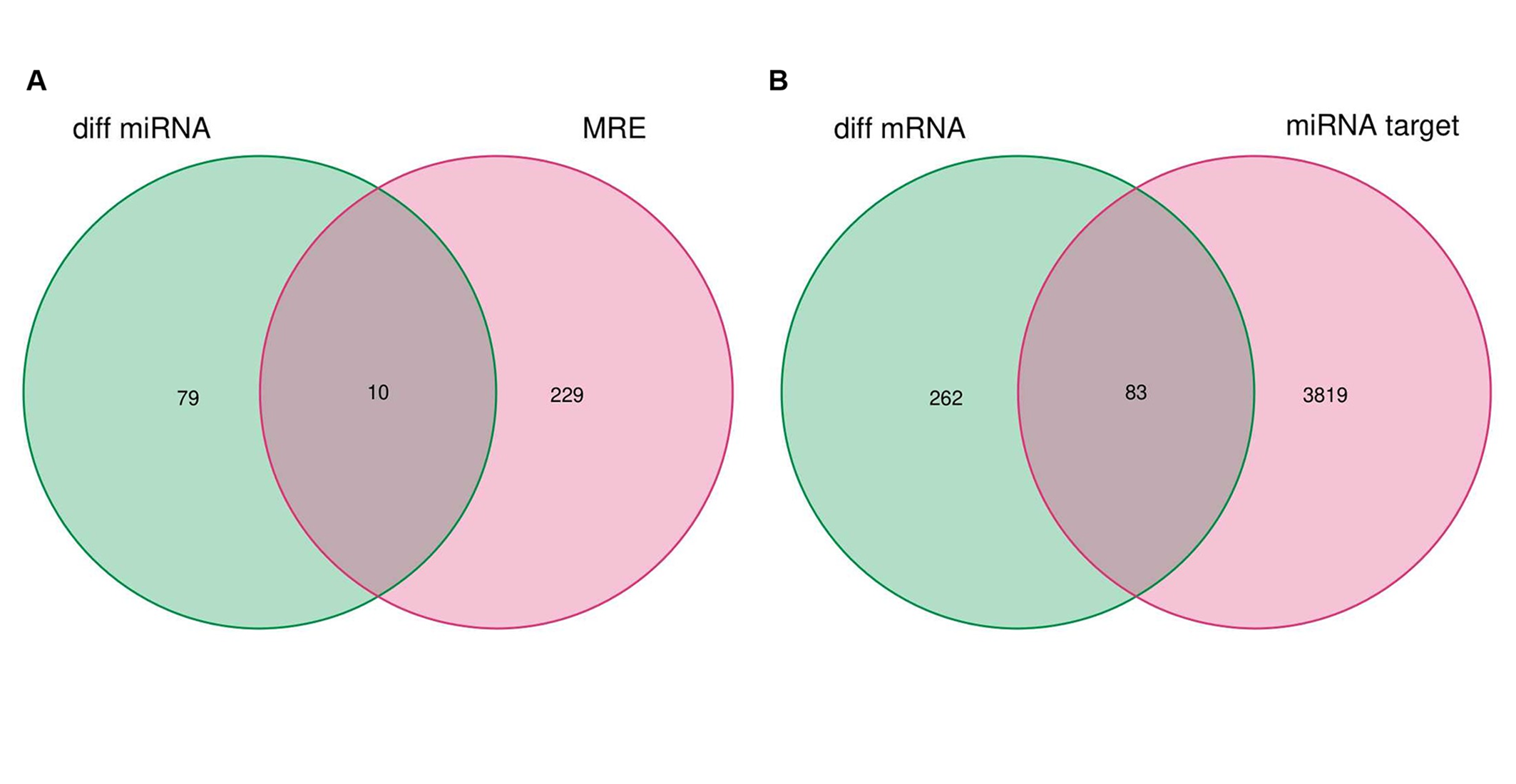

Supplement: Supplementary file 4 [file Image2.TIF]

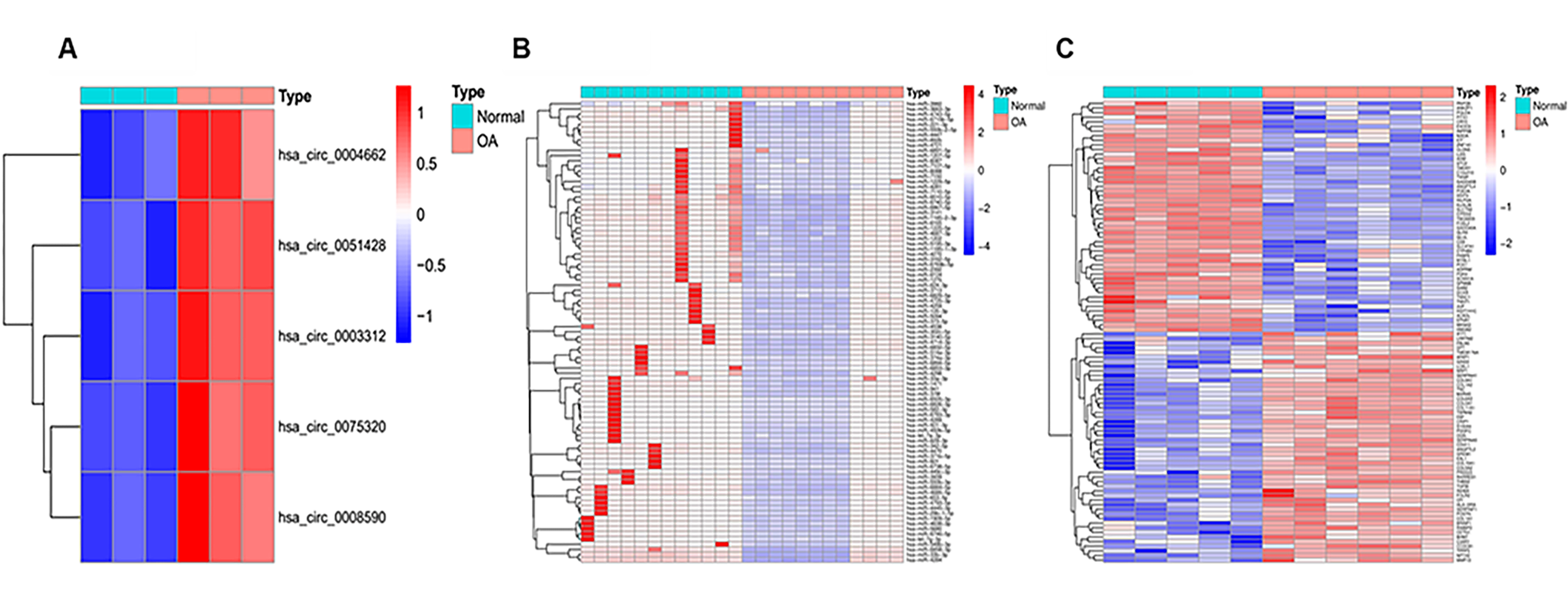

Supplement: Supplementary file 5 [file Image1.TIF]
