# Supplementary material for: Identification of the circRNA–miRNA–mRNA regulatory network in osteoarthritis using bioinformatics analysis
Source: Front Genet. 2022 Sep 16;13:994163. doi: 10.3389/fgene.2022.994163 (PMC9523487; doi:10.3389/fgene.2022.994163)
Supplement: Supplementary file 6 [file Table2.DOCX]

**Table | S2.** Three potential compounds identified by CMap for the treatment of OA

| CMap name | Enrichment score | Target | Mechanism of action |
| --- | --- | --- | --- |
| Noscapine | -99.82 | BDKRB2, SIGMAR1 | Bradykinin receptor antagonist, Tubulin inhibitor |
| Diazepam | -99.71 | GABRA1 et al | Benzodiazepine receptor agonist |
| TG100-115 | -99.65 | PIK3CG et al | PI3K inhibitor |

CMap, connectivity map; OA osteoarthritis.
