# Supplementary material for: Identification of the circRNA–miRNA–mRNA regulatory network in osteoarthritis using bioinformatics analysis
Source: Front Genet. 2022 Sep 16;13:994163. doi: 10.3389/fgene.2022.994163 (PMC9523487; doi:10.3389/fgene.2022.994163)
Supplement: Supplementary file 7 [file Table3.DOCX]

**Table | S3.** Network topological characteristics of six hub genes

| Genes | MCODE score | Degree | Betweenness  Centrality | Closeness  Centrality | ShortestPathLength | TopologicalCoefficient |
| --- | --- | --- | --- | --- | --- | --- |
| SERPINH1(Seed) | 5.00 | 6 | 0.03 | 0.58 | 1.73 | 0.51 |
| COL8A2 | 5.00 | 5 | 0.00 | 0.52 | 1.93 | 0.64 |
| COL6A3 | 5.00 | 8 | 0.23 | 0.68 | 1.47 | 0.39 |
| COL5A1 | 5.00 | 9 | 0.31 | 0.71 | 1.40 | 0.35 |
| COL15A1 | 5.00 | 5 | 0.00 | 0.52 | 1.93 | 0.64 |
| COL11A1 | 5.00 | 7 | 0.16 | 0.60 | 1.67 | 0.45 |
